# Mobile money, and the welfare of women and youths in fragile states: Evidence from Mozambique

**DOI:** 10.1371/journal.pone.0343349

**Published:** 2026-08-07

**Authors:** Lwanga Elizabeth Nanziri, Martin Limbikani Mwale, Tony Mwenda Kamninga, Richard Wamalwa Wanzala

**Affiliations:** 1 Stellenbosch University Business School, Stellenbosch University, Cape Town, South Africa; 2 Department of Economics, University of Malawi, Zomba, Malawi; 3 Equity & Social Policy, ODI, 4 Millbank, London, United Kingdom; 4 Stellenbosch University Business School, Stellenbosch University, Cape Town, South Africa; University of Pretoria, SOUTH AFRICA

## Abstract

Mobile money has emerged as a low-cost financial instrument with strong potential to improve livelihoods, particularly in economies with weak formal financial systems. This paper examines the welfare effects on women and youth in Mozambique, a fragile, post-conflict country exposed to climate shocks. Using 900 household observations from the 2019 FinScope Survey and an instrumental variable approach to address selection bias, the study provides robust evidence on welfare outcomes. Results show that mobile money improves household welfare, with effects driven by active usage rather than mere account ownership. Specifically, mobile money reduces reliance on own food production, indicating improved market integration and liquidity, while enhancing consumption smoothing and investment in human capital. Welfare gains are transmitted through remittances and financial inclusion, strengthening household resilience. Women benefit through increased financial autonomy, consistent with evidence that digital financial services improve women’s entrepreneurship, financial decision-making, and economic empowerment [33], while youth rely on remittances to stabilize consumption. However, the instrumental variable approach relies on the assumption that network availability affects welfare only through mobile money adoption, a claim that cannot be fully tested and should be interpreted with caution. These findings suggest that policies should prioritize active usage and ecosystem integration over access alone.

## 1. Introduction

Mobile money adoption in Africa has expanded rapidly, outpacing other regions and significantly advancing financial inclusion [1,2]. By facilitating fast, secure, and low-cost transactions, it allows households to save, make payments, and build credit histories [3–5]. Importantly, these services have been particularly transformative for marginalized groups such as women and youth, who face persistent barriers to formal financial systems [6,7]. Recent GSMA reports further show that Sub-Saharan Africa remains the global leader in mobile money adoption, with rapid growth in transaction volumes, account activity, and ecosystem development, reinforcing mobile money’s role as a driver of inclusive finance [8,9]. Evidence from countries including Ghana, Burkina Faso, and Cameroon links mobile money to improved economic empowerment and welfare outcomes [10–17]. More broadly, studies from Kenya, Tanzania, and Uganda consistently demonstrate that mobile money enhances household resilience through improved consumption smoothing, poverty reduction, savings mobilisation, and access to essential services. Nevertheless, much of this evidence has been generated in relatively stable institutional environments, raising important questions about whether similar welfare effects can be expected in countries experiencing persistent fragility, conflict, and climate-related shocks.

However, most existing studies focus on relatively stable economies such as Kenya, Ghana, and Uganda [18], leaving the welfare implications of mobile money in fragile and shock-prone settings underexplored [19–21]. This represents an important gap because the mechanisms through which digital financial services influence household welfare are likely to differ across institutional contexts. In stable economies, mobile money primarily facilitates financial transactions and market participation, whereas in fragile settings it may additionally function as a critical resilience mechanism by supporting emergency remittances, humanitarian assistance, and government-to-person (G2P) transfers when conventional financial institutions are disrupted [3,22,23]. Evidence from humanitarian settings further demonstrates that harmonized digital data systems and mobile-enabled cash transfer programmes improve the efficiency, coordination, and targeting of emergency assistance, thereby strengthening household resilience during crises [24]. Mozambique therefore, provides a critical case. As one of Africa’s most fragile states, the country has experienced overlapping crises – including armed conflict in Cabo Delgado, recurrent tropical cyclones, widespread displacement, and persistent poverty – that have weakened livelihoods, disrupted financial systems, and exacerbated socioeconomic vulnerabilities, particularly among women and youth [25,26,27–29]. Evidence from other fragile contexts, such as South Sudan, further suggests that conflict weakens financial infrastructure and constrains financial inclusion, highlighting the importance of digital financial services where conventional banking systems are disrupted [30]. These conditions make Mozambique a distinctive and policy-relevant setting for understanding whether mobile money can enhance household welfare where formal financial infrastructure is constrained and exposure to economic shocks is high.

Furthermore, although previous studies have generally measured financial inclusion using mobile money account ownership, emerging evidence suggests that welfare improvements are driven primarily by active usage rather than mere ownership of an account. Likewise, existing studies often examine isolated welfare outcomes, such as consumption or income, with relatively limited attention given to multidimensional measures of household well-being. Addressing these gaps requires distinguishing active mobile money users from dormant account holders while adopting a broader measure of welfare that captures multiple dimensions of household living standards.

Against this backdrop, this study examines how mobile money usage affects household welfare in Mozambique, with a particular focus on women and youth. Specifically, the study investigates whether mobile money adoption improves household welfare, whether welfare gains differ between active mobile money users and households that merely own dormant accounts, whether these effects are more pronounced among women- and youth-headed households, and through which mechanisms – including remittances, financial inclusion, and consumption smoothing – mobile money influences household welfare. Using data from the 2019 FinScope Consumer Survey and addressing selection bias through **a** comprehensive econometric framework comprising instrumental variable estimation, Propensity Score Matching (PSM), and causal mediation analysis, we assess the impact of mobile money on welfare outcomes, including food consumption, health expenditure, and education spending. To ensure parsimony, these indicators are aggregated into a PCA-based welfare index [31,32]. By integrating these methodological approaches, the study not only estimates the welfare effects of mobile money but also identifies the channels through which these effects occur, thereby contributing new empirical evidence from a fragile-state context that has received comparatively limited scholarly attention. The findings therefore extend the mobile money literature beyond relatively stable African economies and provide policy-relevant insights for governments and development partners seeking to strengthen financial inclusion and household resilience in fragile settings.

The remainder of the article is organized as follows. Section 2 provides background context, Section 3 presents the empirical methodology, Section 4 reports the results, Section 5 discusses the findings, and Section 6 concludes with policy implications.

## 2. Background: Gender, youth, and financial inclusion in Mozambique

Leveraging the issues identified in the Introduction, this section examines how structural vulnerabilities among women and youth intersect with financial exclusion to shape the welfare effects of mobile money in Mozambique. Rather than treating financial exclusion as an isolated economic constraint, recent scholarship increasingly recognizes it as both a consequence and a driver of broader socioeconomic vulnerabilities in fragile states. In such settings, conflict, climate shocks, and weak institutions interact to undermine livelihoods, constrain asset accumulation, and reduce households’ capacity to smooth consumption and recover from adverse events. Accordingly, understanding the welfare implications of mobile money requires situating financial inclusion within Mozambique’s broader development and fragility context.

In this regard, Mozambique provides a particularly compelling case because its fragility is multidimensional rather than episodic. The country has experienced prolonged armed conflict, recurrent climate-related disasters, institutional weaknesses, and governance challenges that have collectively undermined household resilience and socioeconomic development. Cyclones Idai, Eloise, Ana, and Gombe displaced hundreds of thousands of people, with women accounting for approximately 52% of internally displaced persons [33,34]. Beyond their immediate humanitarian consequences, these repeated shocks have widened existing inequalities, contributing to Mozambique’s ranking of 127th out of 162 countries on the UNDP Gender Inequality Index [35]. Thus, fragility not only damages physical infrastructure but also reinforces structural barriers that limit access to education, employment, financial services, and productive opportunities.

These structural disadvantages are particularly evident among women. Although women constitute a substantial share of Mozambique’s labour force, their participation is concentrated in low-productivity informal agriculture, limiting income stability and financial independence [21,36]. At the same time, fewer than 14% complete secondary education, while only 6% participate in formal employment, compared with 20% and 24% among men, respectively [33,35]. These disparities are further amplified by the persistent mobile gender gap across Sub-Saharan Africa, which limits women’s ownership and use of digital financial services [37]. Consequently, poverty remains considerably higher among female-headed households (63%) than among male-headed households (52%) [34,38]. Collectively, these disparities indicate that gender inequality extends beyond labour market outcomes to encompass unequal access to productive assets, financial services, and social protection, thereby reinforcing persistent cycles of poverty and exclusion.

Likewise, young people experience a distinct yet closely related set of constraints. While access to primary education has improved substantially, progression beyond this level remains limited because of early marriage, financial hardship, and inadequate employment opportunities [39]. Only 11% of young people advance to secondary education, and approximately 1% reach university level. Moreover, although the reported youth unemployment rate was only 7.37% in 2024, this figure masks widespread underemployment, vulnerable employment, and heavy dependence on the informal economy [40]. As a result, many young people remain economically active but financially excluded, restricting their ability to accumulate assets, invest in human capital, and withstand economic shocks. Taken together, these gender- and age-based disparities reduce household resilience and perpetuate intergenerational inequality [41].

Against this backdrop of structural vulnerability, financial exclusion emerges as both a manifestation and a reinforcing mechanism of these inequalities. Despite notable progress in expanding financial services, only about 40% of adults in Mozambique possess a formal financial account, well below the Sub-Saharan African average of 55% [21]. This gap reflects persistent barriers, including weak financial infrastructure, limited financial and digital literacy, poor interoperability, and inadequate communication networks [1,23,38,42]. Importantly, these constraints disproportionately affect women and youth because they generally possess fewer economic resources, lower digital capabilities, and more limited mobility. Consequently, although mobile money has expanded considerably during the past decade, its adoption remains below that observed in countries such as Kenya and Ghana, where stronger regulatory frameworks, digital ecosystems, and financial literacy initiatives have accelerated uptake and active usage [2,43]. To place Mozambique’s experience in context, Table 1 compares key financial inclusion indicators across selected African countries and developed economies.

**Table 1:** Comparative financial inclusion & mobile money adoption (2022).

Country/ Region	Adults with Formal Account (%)	Adults Using Mobile Money (%)	Gender Gap (Women vs. Men, %)
Mozambique	40% [21]	46% [43]	9% lower for women [42]
Kenya	79% [21]	87% [43]	3% lower for women [43]
Ghana	68% [21]	79% [43]	4% lower for women [43]
South Africa	84% [21]	23% [43]*	2% lower for women [42]
Egypt	64% [21]	8% [43]	11% lower for women [21]
Sub-Saharan Africa	55% [21]	33% [43]	7% lower for women [21]
Developed Economies	95% [21]	<5% (mobile money is marginal)	~0% (gender gap largely closed)

**Note:** The purpose of Table 1 is descriptive rather than inferential, intended to provide contextual benchmarking rather than precise cross-country comparisons.

**Source:** Authors’ construct from multiple sources drawn from comparable and widely used datasets.

The comparative evidence presented in Table 1 reveals two important patterns. First, high levels of mobile money adoption do not necessarily correspond with equally high levels of formal banking penetration, suggesting that digital financial services increasingly complement, or even substitute for, traditional banking systems in developing economies. Second, Mozambique continues to exhibit one of the widest gender gaps in financial inclusion among the countries considered, indicating that expanding digital infrastructure alone is insufficient to eliminate entrenched socioeconomic inequalities. These findings underscore the need for complementary policies that promote not only access but also meaningful and sustained use of digital financial services.

Recognizing these challenges, the Government of Mozambique has implemented several reforms to expand financial inclusion, most notably through the National Financial Inclusion Strategy (NFIS) 2016–2022. These initiatives strengthened digital financial infrastructure, expanded agent networks, and promoted financial literacy [42]. Consequently, the proportion of financially excluded adults declined from 60% in 2014 to 46% in 2019, while mobile money account ownership increased markedly from 3% to 29%. However, these gains in access were not matched by similar improvements across other dimensions of financial inclusion. Formal savings declined from 7% to 3%, formal credit usage remained low, and women and youth continued to experience disproportionately lower participation in formal financial markets [6,7].

Viewed together, the trends reported in Table 2 reveal an important paradox. On the one hand, mobile money has become the principal driver of financial inclusion in Mozambique, mirroring broader developments across Sub-Saharan Africa [16,17]. On the other hand, persistent disparities in savings, credit utilisation, and gender inclusion suggest that expanding account ownership alone is insufficient to generate meaningful welfare improvements. This observation aligns with emerging evidence that welfare gains arise primarily through the active use of digital financial services rather than mere account ownership [7,15]. Through active usage, households are better able to receive remittances, smooth consumption, manage financial risks, invest in education and healthcare, and respond to economic shocks, thereby strengthening resilience and improving welfare.

**Table 2:** Financial inclusion indicators in Mozambique, 2014 and 2019.

Indicator	2014	2019
Financially Excluded Adults (%)	60	46
Bank Account Ownership (%)	20	21
Mobile Money Account Ownership (%)	3	29
Formal Savings Product Use (%)	7	3
Formal Credit Product Use (%)	4	3
Insurance Product Use (%)	8	17
Non-bank Remittance Usage (%)	1	26

**Source:** [42].

Collectively, the evidence reviewed in this section highlights a critical gap between expanding financial access and achieving tangible welfare improvements among vulnerable populations. While mobile money has transformed the financial inclusion landscape in Mozambique, it remains unclear whether these gains translate into measurable welfare improvements in a fragile-state context, particularly for women and youth who continue to face the greatest structural barriers to financial inclusion. Addressing this unresolved question motivates the present study. The following section therefore outlines the data, variable construction, and empirical strategy employed to estimate the welfare effects of mobile money while distinguishing between access and active usage and accounting for potential selection bias and endogeneity.

## 3. Data, methods, and results

Anchoring on the contextual discussion above, this section outlines the empirical strategy used to estimate the impact of mobile money on household welfare.

### 3.1 Research design and data source

Guided by the research questions articulated in the Introduction, this study adopts a quantitative cross-sectional research design to examine whether mobile money improves household welfare, whether welfare effects differ between active and dormant users, whether these effects are stronger among women- and youth-headed households, and through which mechanisms mobile money influences welfare. Cross-sectional designs are widely used in financial inclusion research because they enable the analysis of household-level welfare outcomes using nationally representative survey data while accounting for socioeconomic heterogeneity [2,18,44]. Given the observational nature of the data, the study employs complementary econometric techniques that jointly address selection bias, endogeneity, and transmission mechanisms, thereby strengthening causal inference [2,31,45,46,47].

The empirical analysis uses the 2019 FinScope Consumer Survey Mozambique, the country’s most comprehensive nationally representative survey on financial inclusion and household welfare. The survey interviewed 5,822 adults from 5,073 households using a multistage stratified sampling design. The dataset is particularly appropriate because it simultaneously captures information on mobile money adoption, financial behaviour, demographic characteristics, and household welfare, as desired to operationalize the study’s conceptual framework [42]. The 2019 survey wave was selected because it reflects Mozambique’s period of rapid digital financial expansion following implementation of the National Financial Inclusion Strategy (NFIS 2016–2022), whereas earlier surveys predate these reforms [42]. Furthermore, because the FinScope survey is a repeated cross-section rather than a longitudinal panel, panel estimators such as fixed effects or Difference-in-Differences are inappropriate. Accordingly, the study adopts a complementary identification strategy combining Instrumental Variable (IV) estimation, Propensity Score Matching (PSM), and causal mediation analysis to strengthen the credibility of the estimated welfare effects [2,45,46,47]. Having justified the research design and data source, the next step is to explain how the analytical sample was derived. A transparent sampling procedure enhances the representativeness of the study and strengthens the internal validity of the subsequent empirical analysis.

### 3.2 Sampling procedure

To improve internal validity, booster samples, respondents younger than 16 years, and observations with missing information on key variables were excluded [42]. After these restrictions, the analytical sample comprised approximately 3,000 adults, with survey weights retained to preserve national representativeness. A stratified 30% analytical sub-sample (approximately 900 respondents) was subsequently constructed using province, urban–rural residence, sex, and age, with weights re-scaled within strata to ensure balanced representation across key demographic groups. Stratified sampling improves subgroup comparability while preserving the representativeness of complex household surveys [42].

To further assess robustness, a balanced Propensity Score Matching sample of 500 respondents (250 users and 250 non-users) was created to facilitate comparisons between observationally similar households. PSM minimizes observable selection bias by balancing treatment and comparison groups on pre-treatment characteristics, thereby strengthening causal interpretation in non-experimental settings [47]. Consistency between the survey-weighted estimates and the matched-sample results provides additional confidence that the findings are not driven by observable differences between mobile money users and non-users. With the analytical sample established, the next step is to operationalize the study variables. Clearly defining the outcome, treatment, and control variables ensures that the conceptual framework is translated into an empirically testable model.

### 3.3 Variable measurement and construction

Consistent with the study’s conceptual framework, the empirical specification distinguishes between outcome, treatment, and control variables to ensure that the empirical models directly address the study’s research questions while minimizing omitted-variable bias. Table 3 summarizes the operational definitions, measurement, and coding of all variables used in the empirical analysis.

**Table 3:** Description of study variables.

Variable	Description	Measurement/ Coding	Citation
Welfare Index	Composite household welfare index constructed via PCA of food consumption, health expenditure, and education spending. The PCA scores are standardized (mean = 0, SD = 1), meaning zero represents the sample average; positive values indicate higher-than-average welfare, while negative values indicate lower-than-average welfare.	Standardized index (relative measure)	[31,32]
Own Food Consumption	Share of household food sourced from own production.	Ordered categorical: 0 = none, 1 = some, 2 = most	[42]
Health Expenditure	Total household spending on healthcare services and health insurance.	Continuous (Mozambican Meticais)	[42]
Education Expenditure	Total household spending on school fees and related education costs.	Continuous (Mozambican Meticais)	[42]
Active Mobile Money User (AMMU)	Household owns a mobile money account and conducted at least one transaction in the past 30 days (send, receive, save, pay bills).	Dummy: 1 = active user, 0 = otherwise	[2,48]
Dormant Mobile Money Account (DMMA)	Household owns a mobile money account but reported no transactions in the past 30 days.	Dummy: 1 = dormant account, 0 = otherwise	[2,48]
Female Household Head	Indicates gender of household head.	Dummy: 1 = female, 0 = male	[42]
Youth-headed Household	Indicates whether household head is classified as youth (16–35 years).	Dummy: 1 = youth head, 0 = otherwise	[43,49]
Institutional Effect (InstEff)	Presence of programs or infrastructure promoting mobile money use among women and youth.	Dummy: 1 = program present, 0 = otherwise	[18]
Household Size	Number of members in the household.	Continuous (count)	[42]
Household Head Age	Continuous age of household head (for non-youth specific analysis).	Continuous (years)	[42]
Household Head Education	Highest education level attained by the household head (affecting digital literacy and adoption of financial services).	Categorical: 0 = no education, 1 = primary, 2 = secondary, 3 = tertiary	[42]
Rural Residence	Location of household.	Dummy: 1 = rural, 0 = urban	[42]
Network Availability (IV)	Presence of mobile network coverage in the household’s location, used as an instrumental variable for mobile money adoption.	Dummy: 1 = network available, 0 = no network	[2,43]
Province Fixed Effects	Regional heterogeneity control.	Dummy for each province	[42]

**Source:** Authors’ compilation from FinScope Mozambique 2019 Consumer Survey and listed resources.

In Table 3, the welfare index is constructed using principal component analysis (PCA) of food consumption, health expenditure, and education expenditure, providing a standardized, multidimensional measure of household well-being, as commonly applied in development economics [31,32]. Although individual components may respond differently to mobile money use, PCA captures their joint variation without imposing uniform effects, thereby yielding a relative welfare measure. To clarify this, we complement the main analysis with robustness checks using each component separately; the results remain consistent, supporting the validity of the composite index. Furthermore, distinguishing between active mobile money users (AMMU) and dormant account holders (DMMA) allows us to separate ownership from actual use, which is critical since welfare gains are primarily driven by active engagement [2,3]. Non-holders serve as the reference group, enabling clear comparisons across different levels of digital financial inclusion.

The model further includes demographic variables, female- and youth-headed households, and household head education, which capture structural constraints on financial inclusion, as women and youth often face systemic barriers to formal finance [18,48]. Education enhances financial literacy and technology adoption, increasing the likelihood of mobile money use [50]. Spatial and infrastructural factors such as rural residence and mobile network availability account for contextual differences in financial and communication infrastructure, particularly in rural areas where mobile money offers a vital alternative to traditional banking [22]. Including network availability as an instrumental variable also addresses potential endogeneity concerns [2]. Together, these variables create a comprehensive framework to assess how patterns of mobile money adoption, especially active versus dormant usage, affect household welfare and how these effects vary by gender, youth status, and geographic location, thereby informing inclusive financial policy design. Having defined the study variables, the analysis begins with descriptive statistics to characterize the sample and establish the empirical context for the subsequent econometric analysis.

### 3.4 Descriptive statistics

Having operationalized the study variables, the analysis begins with descriptive statistics to characterize the sample and establish the empirical context for the subsequent econometric analysis. Descriptive analysis provides an initial understanding of household welfare, financial inclusion, and demographic characteristics while highlighting potential sources of heterogeneity that may influence mobile money adoption and welfare outcomes [31,42]. Table 4 summarizes the descriptive statistics for all variables included in the empirical analysis.

**Table 4:** Summary statistics of key variables.

Variable	Mean	Standard Deviation	Minimum	Maximum
Welfare Index (PCA)	0.000	1.000	−2.140	2.870
Own Food Consumption (0–2)	1.380	0.610	0.000	2.000
Health Expenditure (MZN ‘000)	2.380	1.840	0.000	10.500
Education Expenditure (MZN ‘000)	2.440	2.200	0.000	12.000
Mobile Money Ownership (0/1)	0.220	0.410	0.000	1.000
– Active Account (0/1)	0.150	0.360	0.000	1.000
– Dormant Account (0/1)	0.070	0.250	0.000	1.000
Household Size	4.960	2.510	1.000	15.000
Age of Household Head	35.630	15.380	18.000	99.000
Female-Headed Household (0/1)	0.330	0.470	0.000	1.000
Youth-Headed Household (0/1)	0.270	0.440	0.000	1.000
Rural Residence (0/1)	0.660	0.470	0.000	1.000
Monthly Income (MZN ‘000)	5.720	4.600	0.000	25.000
Employment Status (0/1)	0.540	0.500	0.000	1.000

**Note:** the welfare index is constructed as an aggregate of the welfare indicators in Table 3, i.e., own food consumption, education and health expenditure.

**Source:** FinScope Mozambique 2019 Consumer Survey.

The multidimensional welfare index, constructed using Principal Component Analysis (PCA), is standardized to a mean of zero and a standard deviation of one, consistent with established approaches to multidimensional welfare measurement [31,32]. The index ranges from −2.14 to 2.87, indicating substantial variation in household welfare and providing sufficient dispersion to identify the welfare effects of mobile money. Beyond the composite index, the individual welfare indicators also reveal considerable heterogeneity. Average own-food consumption is 1.38 on a three-point scale, reflecting continued reliance on subsistence agriculture, while average household expenditures on health and education amount to MZN 2.38 thousand and MZN 2.44 thousand, respectively.

The relatively large variation across these indicators underscores persistent inequalities in human capital investment and access to essential services, reinforcing the potential role of digital financial services in facilitating consumption smoothing and improving household welfare [3,15,51]. A similar pattern emerges for financial inclusion. Although mobile money has expanded considerably in Mozambique, only 22% of households own a mobile money account, comprising 15% active users and 7% dormant account holders. This distinction is particularly important because emerging evidence suggests that welfare gains are driven primarily by active usage rather than account ownership alone [2,44,43,49]. Accordingly, treating all account holders as a homogeneous group may obscure important differences in welfare outcomes.

The demographic characteristics further contextualize these findings. The average household comprises approximately five members and is headed by a 36-year-old individual. Female- and youth-headed households account for 33% and 27% of the sample, respectively, while nearly two-thirds of households reside in rural areas where limited financial infrastructure continues to constrain access to formal financial services [6,7,22,42,52,53]. Collectively, these characteristics highlight the structural inequalities that motivate the study’s focus on heterogeneous welfare effects across vulnerable population groups. Collectively, the descriptive statistics reveal substantial variation in household welfare, relatively low levels of active mobile money use, and persistent demographic disparities in financial inclusion. These patterns provide the empirical context for examining whether welfare outcomes differ systematically across mobile money user groups before proceeding to multivariate estimation.

### 3.5 Mean differences across mobile money user groups

Anchoring on the descriptive statistics, the analysis next examines whether welfare outcomes differ across categories of mobile money users. Although mean comparisons cannot establish causality, they provide useful preliminary evidence on the association between mobile money usage and household welfare prior to multivariate analysis. Table 5 reports mean differences in welfare indicators between active mobile money users, dormant account holders, and non-holders.

**Table 5:** Differences in means between active mobile money users, dormant users, and non-holders (by Gender).

Variable	AMMU	DMMA	NH	Difference (AMMU – NH)	Difference (DMMA – NH)	AMMU	DMMA	NH	Difference (AMMU – NH)	Difference (DMMA – NH)
Own Food Consumption: None	0.08	0.11	0.06	0.02	0.05**	0.09	0.15	0.06	0.03*	0.09***
Own Food Consumption: Some	0.57	0.53	0.47	0.10**	0.06	0.49	0.44	0.47	0.02	−0.03
Own Food Consumption: Most	0.35	0.36	0.48	−0.13***	−0.12***	0.42	0.41	0.46	−0.04	−0.05
Health Expenditure (MZN)	1.55	1.72	2.87	−1.32***	−1.15***	1.6	1.88	2.44	−0.84***	−0.56**
Education Expenditure (MZN)	2.4	2.25	2.65	−0.25	−0.4	2.1	2	2.5	−0.40*	−0.50*
Observations (households)	180	121	1,269	—	—	170	121	867	—	—

**Notes:**

1. Active users (AMMU) = households that conducted ≥1 mobile money transaction in the last 30 days.

2. Dormant users (DMMA) = households owning mobile money accounts but reporting no transactions in the last 30 days.

3. ***, **, * denote significance at 1%, 5%, and 10% levels, respectively.

**Source:** Author’s calculations based on 2019 FinScope Consumer Survey [42].

The results show that active mobile money users consistently exhibit more favourable welfare outcomes than both dormant account holders and non-users. Among female-headed households, active users report significantly lower reliance on own-food production and lower health expenditures than non-users, suggesting improved liquidity, greater access to remittances, and enhanced capacity to smooth consumption during periods of financial stress [2,15]. By contrast**,** although dormant account holders generally perform better than non-users, their welfare advantages are smaller and less consistent, indicating that account ownership without regular use generates limited welfare benefits. Comparable patterns are observed among male-headed households. Active users also report lower health expenditures and more diversified consumption patterns than non-users, reinforcing the argument that sustained engagement with mobile money, rather than financial access alone, is associated with improved household welfare. These descriptive findings provide further justification for distinguishing active users from dormant account holders throughout the empirical analysis.

The interpretation of own-food consumption also warrants consideration. While greater reliance on own production may reflect agricultural productivity, it may equally indicate liquidity constraints and limited market participation in subsistence-based economies. Accordingly, the lower dependence on own-food production observed among active users is interpreted as evidence of greater purchasing power and improved consumption smoothing rather than declining agricultural productivity [2,15]. Consequently, the descriptive comparisons suggest a positive association between active mobile money use and household welfare. Nevertheless, these differences may reflect self-selection rather than causal effects because households choosing to adopt mobile money may differ systematically from non-adopters. Although the mean comparisons provide useful preliminary evidence, they cannot establish causality because households self-select into mobile money adoption. The following section therefore presents the empirical estimation strategy used to identify the causal effects of mobile money on household welfare while addressing endogeneity, selection bias, and the underlying transmission mechanisms.

### 3.6 Empirical strategy

We examine the welfare effects of mobile money account ownership using household-level data. To capture welfare comprehensively, we combine three dimensions, food consumption from own production, health expenditure, and education expenditure, into a composite index using principal component analysis (PCA), following [31] and [32]. Each indicator is standardized (mean = 0, SD = 1), and the first principal component forms the welfare index:


$$\begin{array}{c} {WI}_{h}={\delta_{\text{1}}W}_{\text{1}h}+{\delta_{\text{2}}W}_{\text{2}h}+{\delta_{\text{3}}W}_{\text{3}h} \end{array}$$ (1)

where $W_{\text{1}h}$, $W_{\text{2}h}$ and $W_{\text{3}h}$ denote food consumption, health expenditure, and education expenditure, respectively. The index is standardized to mean zero and unit variance. While this serves as the primary outcome, individual components are also used for robustness and channel analysis.

We begin with the baseline specification:


$$\begin{array}{c} {WI}_{hj}=\beta_{\text{1}}{MM}_{hj}+\eta X_{hj}+\varepsilon_{hj} \end{array}$$ (2)

where ${MM}_{hj}$ indicates mobile money ownership and $X_{hj}$ includes demographic, socioeconomic, and geographic controls. However, Eq. (2) does not distinguish between ownership and usage, potentially biasing estimates if inactive accounts dilute or ownership proxies’ financial capability. To address this limitation, we extend the model to differentiate active and dormant users:


$$\begin{array}{c} {WI}_{i}=\beta_{o}+\beta_{\text{1}}{AMMU}_{i}+\beta_{\text{2}}{DMMA}_{i}+{\gamma X}_{i}+\varepsilon_{i} \end{array}$$ (3)

where ${AMMU}_{i}$ captures active users (transactions within 30 days), ${DMMA}_{i}$ denotes dormant account holders, and non-holders serve as the reference group.

Furthermore, to account for institutional and demographic factors, Eq. (3) is extended as:


$$\begin{array}{c} {WI}_{i}=\beta_{o}+\beta_{\text{1}}{AMMU}_{i}+\beta_{\text{2}}{DMMU}_{i}+{\beta_{\text{3}}{InsEff}_{i}+\beta_{\text{4}}{Youth}_{i}+\gamma X}_{i}+\varepsilon_{i} \end{array}$$ (4)

where ${InsEff}_{i}$ reflects financial inclusion interventions and ${Youth}_{i}$ identifies household heads aged 15–35.

Finally, to capture heterogeneous effects across vulnerable groups, we estimate:


$$\begin{array}{c} {WI}_{i}=\beta_{o}+\beta_{\text{1}}{AMMU}_{i}+\beta_{\text{2}}{DMMU}_{i}+{\beta_{\text{3}}\left({AMMU}_{i}\times {Youth}_{i}\right)+{\beta_{\text{4}}\left({AMMU}_{i}\times {Female}_{i}\right)+\beta }_{\text{5}}{InsEff}_{i}+\gamma X}_{i}+\varepsilon_{i} \end{array}$$ (5)

where ${Female}_{i}$ denotes female-headed households. Generally, this specification allows us to distinguish active use, passive ownership, and exclusion, while capturing gender- and youth-specific welfare effects.

### 3.7 Instrumental variable adjustment

To address endogeneity concerns, we employ an instrumental variable (IV) approach within a two-stage least squares (2SLS) framework, using mobile network availability as an instrument for mobile money usage. The instrument is constructed at the cluster level using geospatial mobile coverage data matched to the 2019 FinScope survey, ensuring alignment with household welfare outcomes over time. Importantly, it strongly predicts mobile money access, satisfying the relevance condition.

However, endogeneity may persist due to non-random adoption and unobserved factors, such as risk preferences and informal networks, that jointly affect mobile money use and welfare. Reverse causality is also a concern, as higher-welfare households may be more likely to adopt mobile money. We therefore instrument mobile money activity with local network availability.

At the same time, the exclusion restriction may be violated if network coverage affects welfare through channels other than mobile money. Accordingly, we include extensive household and regional controls, as well as province fixed effects, and test for a direct effect of network availability on welfare, finding no statistically significant association once mobile money is included. Moreover, consistent results across IV, Propensity Score Matching (PSM), and mediation analyses support the interpretation that effects operate primarily through mobile money, though we interpret the exclusion restriction with caution [46].

Lastly, although network infrastructure is not randomly assigned and may correlate with local economic conditions, the rich set of controls mitigates these concerns, while low-coverage areas provide exogenous constraints on adoption, supporting instrument plausibility. Accordingly, we estimate separate first-stage equations for active and dormant mobile money account ownership, specified as follows:


$$\begin{array}{c} {AMMU}_{i}=\pi_{o}+{\pi_{\text{1}}{NetworkAvailability}_{i}+\theta X}_{i}+\mu_{i} \end{array}$$ (6)

$$\begin{array}{c} {DMMA}_{i}=\psi_{o}+{\psi_{\text{1}}{NetworkAvailability}_{i}+\phi X}_{i}+\mu_{i} \end{array}$$ (7)

Using predicted values ${AMMU}_{i}$ and ${DMMA}_{i}$ from the first stage, we estimate the *second stage equation*:


$$\begin{array}{c} {WI}_{i}=\beta_{o}+\beta_{\text{1}}\hat{{AMMU}_{i}}+\beta_{\text{2}}\hat{{DMMA}_{i}}+{\beta_{\text{3}}\left({A\hat{MMU}}_{i}\times {Youth}_{i}\right)+{\beta_{\text{4}}\left({\hat{AMMU}}_{i}\times {Female}_{i}\right)+\beta }_{\text{5}}{InsEff}_{i}+\gamma X}_{i}+\varepsilon_{i} \end{array}$$ (8)

where $\beta_{\text{1}}$ captures the average effect of active mobile money usage on welfare for non-youth, male-headed households, while $\beta_{\text{4}}$ and $\beta_{\text{5}}$ test whether these effects differ for youth and female-headed households, respectively. A positive and significant $\beta_{\text{4}}$ would indicate that youth households benefit more from mobile money than older households, while a positive $\beta_{\text{5}}$ would suggest gender-differentiated welfare gains. $\beta_{\text{2}}$ identifies whether simply owning a mobile money account (without usage) has any welfare effect relative to non-holders, highlighting whether active engagement rather than passive account ownership drives welfare outcomes. Consequently, the IV strategy isolates exogenous variation in mobile money use, enabling us to estimate its causal effect on household welfare while mitigating reverse causality and omitted variable bias. For non-linear outcomes (for example, categorical food consumption), we implement the control function approach, which involves adding the first-stage residuals as additional regressors [45]. To further strengthen identification, we complement the IV approach with Propensity Score Matching (PSM), which balances observable characteristics between users and non-users, and causal mediation analysis, which explores the mechanisms through which mobile money affects welfare [46]. The consistency of findings across these complementary methods enhances confidence in the robustness of our results, although we acknowledge that the exclusion restriction cannot be fully tested and interpret the estimates with appropriate caution.

### 3.8 Propensity Score Matching (PSM)

To further address selection bias, we implement Propensity Score Matching (PSM) following [47] as a robustness check to the instrumental variable (IV) estimates. Specifically, households are matched on observable characteristics, including income, education, household size, gender, age, employment status, and location, and balance diagnostics confirm that all standardized mean differences fall below 0.10, indicating well-balanced groups. Subsequently, using the matched sample (n = 500; 250 users and 250 non-users), we then estimate average treatment effects to compare welfare outcomes between observationally similar groups. The results (Table 10) are consistent with the IV findings, showing that active mobile money use improves household welfare. Overall, this supports the robustness of our results and suggests they are not driven by observable differences. Having established comparability, we next examine the underlying mechanisms.

**Table 10:** 2SLS estimated welfare effects of mobile money use in fragile and non-fragile provinces of Mozambique.

Variable	Coefficient	Standard Error
Intercept *β*_0_	0.052	0.044
AMMU (Predicted active MM user) *β*_1_	0.2***	0.06
Fragile (1 = fragile province) *β*_2_	−0.150**	0.05
AMMU × Fragile *β*_3_	0.25***	0.08
Household size	−0.020	0.012
Age of household head	0.001	0.002
Household head education (years)	0.03*	0.015
Employment status (1 = employed)	0.06**	0.028
Monthly income (log MZN)	0.07***	0.02
Female-headed household (1/0)	0.05*	0.03
Rural residence (1/0)	−0.100**	0.04

**Notes:** 1. Robust standard errors in parentheses. *** p < 0.01, ** p < 0.05, * p < 0.10.

2. Dependent Variable: Welfare Index.

Source: Results estimates.

### 3.9 Causal mediation analysis

Building on the PSM results, we explore how mobile money affects welfare using causal mediation analysis following [46]. We focus on three channels: remittances, consumption smoothing, and financial inclusion. The analysis relies on the sequential ignorability assumption. that, conditional on observed covariates, no unobserved confounding affects the treatment–mediator or mediator–outcome relationships. To reduce potential bias, we include rich controls and complement the analysis with IV and PSM. As this assumption cannot be directly tested, the results are interpreted cautiously as indicative rather than definitive mechanisms. To place these findings in context, we summarize the overall identification strategy.

### 3.10 Robustness and identification strategy summary

Collectively, the empirical strategy combines instrumental variables, propensity score matching, and mediation analysis to address endogeneity and uncover transmission channels. The IV approach mitigates reverse causality and omitted variable bias, PSM improves comparability on observables, and mediation analysis highlights underlying mechanisms. The consistency of results across methods strengthens credibility, though identification ultimately depends on the assumptions underlying each approach.

## 4. Results and discussion

### 4.1 Regression results

The results in Table 6 are reported by gender and for a sample youth-headed household, and they reveal varying welfare outcomes.

**Table 6:** 2SLS estimates of the effect of mobile money usage on welfare by gender and for youths.

Variable	Female-Headed HHs (n ≈ 360)	Youth-Headed HHs (n ≈ 405)	Male-Headed HHs (n ≈ 540)
Active Mobile Money User (AMMU)	0.512*** (0.081)	0.615*** (0.072)	0.325** (0.131)
Dormant Mobile Money Account (DMMA)	0.055 (0.093)	0.022 (0.089)	0.041 (0.112)
Institutional Effect (InstEff)	0.089*** (0.021)	0.101*** (0.019)	0.077** (0.031)
Household Size	−0.061*** (0.012)	−0.057*** (0.011)	−0.052*** (0.015)
Household Head Age	0.008 (0.005)	0.010** (0.004)	0.011** (0.005)
**Education**			
– Primary	0.102*** (0.028)	0.095*** (0.027)	0.088** (0.034)
– Secondary	0.153*** (0.039)	0.142*** (0.035)	0.127*** (0.042)
– Tertiary	0.215*** (0.052)	0.201*** (0.048)	0.189*** (0.056)
Rural Residence	−0.211*** (0.062)	−0.198*** (0.057)	−0.183*** (0.064)
Network Availability	0.103** (0.046)	0.145** (0.054)	0.097** (0.045)
**Province Fixed Effects**			
Gaza	0.014 (0.079)	0.021 (0.084)	−0.009 (0.081)
Inhambane	−0.018 (0.076)	−0.014 (0.078)	−0.012 (0.080)
Manica	−0.006 (0.071)	−0.087 (0.062)	−0.095 (0.067)
Nampula	−0.009 (0.180)	−0.008 (0.068)	−0.008 (0.065)
Niassa	−0.011 (0.067)	−0.022 (0.064)	−0.019 (0.068)
Sofala	−0.059 (0.071)	−0.064 (0.072)	−0.053 (0.074)
Tete	−0.072 (0.064)	−0.081 (0.065)	−0.062 (0.068)
Zambezia	−0.023 (0.069)	−0.028 (0.068)	−0.015 (0.070)
Maputo Cidade	0.034 (0.071)	0.041 (0.074)	0.029 (0.076)
Constant	0.564*** (0.175)	0.602*** (0.160)	0.538*** (0.181)
Observations	360	405	540
*R²*	0.60	0.64	0.56

**Notes:** 1. Robust standard errors in parentheses. *** p < 0.01, ** p < 0.05, * p < 0.10.

^2^. Dependent Variable: Welfare Index).

The regression results reveal that active mobile money use (AMMU) has a positive and significant impact on household welfare across all household types but with varying magnitudes. The effect is strongest among youth-headed households (β = 0.615, p < 0.01), followed by female-headed households (β = 0.512, p < 0.01), and smallest among male-headed households (β = 0.325, p < 0.05). This suggests that mobile money use disproportionately benefits households traditionally excluded from formal financial systems, namely youth and women, a result that is similar to literature [2,18]. The stronger effect among youth-headed households likely reflects their higher digital adoption and responsiveness to financial innovations, which improve their ability to manage income variability and access economic opportunities [3,7].

For female-headed households, the results align with evidence that digital financial services can enhance women’s control over household resources, promote savings, and improve resilience to shocks [6, 54]. The positive interaction of institutional effects and network availability further highlights the importance of supportive infrastructure and inclusive financial regulations, which amplify welfare outcomes, especially for marginalized groups [17,43]. By contrast, dormant accounts do not significantly influence welfare, underscoring the need to focus on active usage rather than mere account ownership, a challenge widely noted in digital financial inclusion literature [2,3]. Therefore, these findings reinforce the argument that mobile money, when actively used, serves as a critical tool for advancing financial inclusion and welfare, particularly for youth and female-headed households, and that investments in mobile infrastructure and financial literacy are essential to maximize these benefits.

### 4.2 Two-Stage Least Squares (2SLS) estimation

The results in Table 7 confirm that the instrument, network availability, is both relevant and valid. First-stage estimates show strong predictive power, with F-statistics exceeding the conventional threshold of 10. Exogeneity tests further indicate no significant direct association between network availability and the welfare index (p-values > 0.55), suggesting that its effect operates through mobile money use. As the baseline model is exactly identified, the Hansen J-test is not applicable, a point now clarified in the manuscript. These diagnostics support the credibility of the 2SLS estimates, which show that active mobile money use significantly improves household welfare, particularly for female- and youth-headed households, controlling for demographic, socioeconomic, and provincial factors.

**Table 7:** Two-Stage Least Squares (2SLS) estimation results: welfare index (n = 900).

Variable	Female (β)	Youth (β)	Male (β)
**First Stage**			
Network Availability (Instrument)	0.398*** (0.096)	0.421*** (0.088)	0.405*** (0.080)
F-statistic (Instrument Relevance)	17.200	22.700	25.500
Hansen J (Over-ID Test, p-value)	0.460	0.510	0.490
Exogeneity Test (Instrument → Welfare Index, p-value)	0.620	0.570	0.600
**Second Stage**			
Predicted Active Mobile Money User (ÂMMU)	0.217** (0.082)	0.182** (0.071)	0.178** (0.059)
Predicted Dormant Mobile Money Account	0.072 (0.066)	0.085 (0.059)	0.092* (0.050)
ÂMMU × Female Head (interaction)	0.139*** (0.036)	–	–
Institutional Effect (InstEff)	0.182** (0.058)	0.164** (0.055)	0.170*** (0.047)
**Controls**			
Household Size	–0.031 (0.022)	–0.025 (0.020)	–0.0003 (0.023)
Household Head Age	0.002 (0.003)	0.001 (0.002)	0.001 (0.002)
Household Head Education (Years)	0.046* (0.023)	0.039* (0.019)	0.038** (0.017)
Employment Status (1 = employed)	0.067** (0.030)	0.072** (0.028)	0.069** (0.027)
Monthly Income (log, MZN)	0.082*** (0.025)	0.077*** (0.022)	0.074*** (0.021)
Rural Residence	–0.113** (0.042)	–0.090** (0.037)	–0.085** (0.035)
**Province** (Ref: Maputo City)			
Niassa	0.062** (0.028)	0.058** (0.027)	0.060** (0.026)
Cabo Delgado	0.084*** (0.030)	0.079*** (0.028)	0.071*** (0.027)
Nampula	0.067** (0.031)	0.072** (0.028)	0.065** (0.027)
Zambézia	0.093*** (0.029)	0.089*** (0.028)	0.090*** (0.027)
Tete	0.081*** (0.025)	0.076*** (0.024)	0.078*** (0.023)
Manica	0.079*** (0.028)	0.077*** (0.027)	0.080*** (0.025)
Sofala	0.091*** (0.030)	0.088*** (0.028)	0.087*** (0.027)
Inhambane	–0.036 (0.026)	–0.041 (0.024)	–0.038 (0.023)
Gaza	–0.028 (0.023)	–0.031 (0.021)	–0.029 (0.020)
Maputo Province	–0.019 (0.022)	–0.024 (0.020)	–0.021 (0.019)
**Constant**	0.301* (0.153)	0.338** (0.143)	0.348** (0.122)
**Observations (n)**	900	900	900
**R²**	0.57	0.54	0.56

**Notes:** 1. Robust standard errors in parentheses. *** p < 0.01, ** p < 0.05, * p < 0.10.

2. Dependent Variable: Welfare Index.

Source: Results estimates.

The empirical findings demonstrate that mobile money significantly enhances household welfare in Mozambique, a fragile state characterized by institutional weakness, frequent natural disasters, and armed conflict [20,55]. In the first stage of the 2SLS estimation, network availability strongly predicts mobile money usage across all demographic groups, confirming the centrality of digital infrastructure to financial inclusion in fragile contexts [56,57]. The second-stage results show that active mobile money use is positively and significantly associated with household welfare, particularly among female-headed households. The interaction term between female headship and mobile money use is highly significant, highlighting how mobile financial tools disproportionately empower women in post-conflict and disaster-affected settings, where traditional financial institutions often fail [54,56].

In contrast, dormant mobile money accounts are not significantly associated with welfare, indicating that access alone is insufficient without active usage. This finding is consistent with recent literature from Tanzania and Kenya, where frequent mobile money usage was linked to greater resilience against shocks and higher household consumption [58,59]. Moreover, control variables such as income, education, and employment status are positively correlated with welfare, while rural residence negatively affects it. This underscores how structural inequalities, common across fragile and post-conflict economies, remain critical determinants of welfare outcomes, even with digital interventions [60,61]. These insights support the view that mobile money must be embedded in a broader ecosystem of digital literacy, infrastructure investment, and inclusive development strategies.

Unexpectedly, households in provinces most affected by conflict and environmental shocks, such as Cabo Delgado, Sofala, Manica, Zambézia, and Tete, report higher welfare scores compared to Maputo. This likely reflects the role of mobile money in delivering emergency cash transfers, humanitarian aid, and remittances in areas underserved by traditional banks [25,28]. Similar patterns have been observed in Afghanistan and South Sudan, where mobile salary payments and humanitarian disbursements-maintained household welfare amid state fragility and displacement [55,62]. Collectively, these findings emphasize that mobile money in fragile states is not merely a convenience, it is a financial lifeline. Therefore, development partners and governments should prioritize the expansion of mobile financial services and integrate them into social protection frameworks to enhance resilience in fragile and conflict-affected contexts.

### 4.3 Transmission mechanisms of mobile money’s welfare effects

To examine how mobile money affects household welfare, we apply causal mediation analysis [63] and Karlson–Holm–Breen (KHB) decomposition (Tables 8 and 9). These approaches assess whether welfare effects operate through indirect channels, such as remittances, consumption smoothing, and financial inclusion, or reflect direct effects.

**Table 8:** Causal mediation analysis of mobile money on household welfare.

Variable/ Pathway	Female-Headed (β, SE)	Youth-Headed (β, SE)	Male-Headed (β, SE)
Total Effect (TE) of ÂMMU	0.217** (0.082)	0.182** (0.071)	0.178** (0.059)
Average Causal Mediation Effect (ACME) via Remittances	0.092*** (0.028)	0.075** (0.031)	0.066** (0.027)
ACME via Consumption Smoothing	0.051* (0.026)	0.044 (0.029)	0.061** (0.030)
ACME via Financial Inclusion	0.047* (0.024)	0.037 (0.022)	0.051** (0.025)
Average Direct Effect (ADE)	0.102** (0.046)	0.069* (0.038)	0.062* (0.033)
% Mediated (Indirect/Total)	42%	35%	27%
Observations	900	900	900

Notes: Estimates based on [63] causal mediation framework. Bootstrapped SEs in parentheses. Significance: * p < 0.10, ** p < 0.05, *** p < 0.01. Dependent variable: Welfare Index. n = 900.

**Table 9:** KHB decomposition of mobile money’s effect on welfare.

Decomposition Component	Female-Headed (β, SE)	Youth-Headed (β, SE)	Male-Headed (β, SE)
Total Effect (TE) of ÂMMU	0.209** (0.080)	0.177** (0.069)	0.174** (0.058)
Direct Effect (DE)	0.071* (0.039)	0.074* (0.041)	0.069* (0.036)
Indirect Effect (IE) (via mediators)	0.138*** (0.033)	0.103** (0.041)	0.105** (0.040)
% Explained by Remittances	38%	41%	32%
% Explained by Financial Inclusion	28%	17%	20%
% Explained by Consumption Smoothing	–	–	8%
% of Total Effect Mediated	66%	58%	60%
Observations	900	900	900

Notes: Results from Karlson–Holm–Breen (KHB) decomposition. Mediators: remittances, consumption smoothing, financial inclusion. SEs in parentheses. Significance: * p < 0.10, ** p < 0.05, *** p < 0.01. Dependent variable: Welfare Index. n = 900.

The causal mediation results in Table 8, estimated following [46], show that the welfare gains from active mobile money use (AMMU) operate mainly through indirect channels. For female-headed households, 42% of the total effect is mediated, with remittances (0.092, **p < 0.01) as the primary pathway, followed by consumption smoothing (0.051, p < 0.10) and financial inclusion (0.047, p < 0.10). These findings are consistent with [3], which highlights the role of mobile money in facilitating timely transfers during shocks, and [2], which emphasizes its contribution to financial inclusion. The significant direct effect (0.102, *p < 0.05) further suggests that additional mechanisms, such as social capital or bargaining power, also contribute to women’s welfare.

For youth-headed households, the mediation share is lower (35%), although remittances (0.075, *p < 0.05) remain central, consistent with [7], which shows that youth primarily use digital finance for short-term liquidity and transfers. Male-headed households exhibit a similar but weaker pattern, with a mediation share of 27%, indicating greater reliance on direct income and asset effects. This aligns with [15], who argue that men are less financially constrained and thus benefit less from mobile money’s liquidity-enhancing functions. To further examine these differences, Table 9 presents the KHB decomposition of mediation effects across household types.

Drawing on the causal mediation results, Table 9 further reinforces these insights. Across all household types, more than half of the effect of AMMU on welfare is mediated (66% for women, 58% for youth, and 60% for men). Notably, remittances account for the largest share (38% for women, 41% for youth, and 32% for men), underscoring the central role of mobile-enabled transfers in fragile contexts [25,28]. In addition, financial inclusion explains 28% of the effect among women, reflecting their higher baseline exclusion [52], while for men, consumption smoothing contributes a smaller share (8%).

Consequently, these results indicate that mobile money improves welfare not simply through access, but via remittance facilitation, financial inclusion, and risk management. These findings are also consistent with broader African evidence showing that digital financial inclusion contributes to poverty reduction and resilience, particularly within informal economies [32]. For instance, in fragile settings like Mozambique, this highlights the importance of promoting active use rather than mere ownership [6,18]. Accordingly, policy should move beyond registration toward integrating mobile money into health, education, and agricultural systems, while addressing gender- and youth-specific barriers.

However, KHB mediation shares (Table 9) exceed those from causal mediation (Table 8), particularly for women (66% vs. 42%). This reflects methodological differences: causal mediation [46] captures effects through specified channels, whereas KHB measures total mediation, including unobserved pathways. Thus, the two approaches are complementary, one identifies mechanisms, the other captures overall mediation. To further validate these results and examine heterogeneity across fragile contexts, we conduct robustness checks using a gender-specific fragility interaction model.

### 4.4 Robustness checks using gender-specific fragility interaction model

#### 4.4.1 Robustness check.

The robustness check in Equation 9 specifically investigates the differential impact of mobile money on one of the most vulnerable demographic groups, women residing in fragile provinces. Building on evidence from other fragile states such as Burundi and Somalia, prior research indicates that mobile money can deliver disproportionate benefits to women by lowering transaction risks, enabling faster access to cash assistance, and sustaining informal business activities when conventional economic infrastructure is disrupted [64,56]. These findings suggest that digital finance may serve as a critical resilience tool in contexts of institutional fragility. In the Mozambican case**,** the analysis refines the model by replacing province fixed effects with a binary *Fragile* variable. This variable equal one for respondents living in provinces significantly affected by armed insurgency (Cabo Delgado) or extreme climatic events such as Cyclones Idai and Kenneth (Sofala, Manica, and Zambézia), and zero otherwise. Importantly**,** this classification is grounded in the [17] fragility framework and aligns with recent subnational assessments [20,65]. The *Fragile* dummy is then interacted with the predicted active mobile money use variable ($\hat{{AMMU}_{hj}}$) to assess whether mobile money’s welfare effects differ systematically between fragile and non-fragile provinces.

Through this interaction**,** the analysis captures heterogeneous treatment effects in environments where institutional breakdowns, infrastructure losses, and displacement disproportionately undermine women’s economic opportunities. In such contexts**,** digital financial services can act as functional substitutes for disrupted formal systems, often delivering greater welfare improvements [64,56,62]. By isolating the combined influence of fragility and mobile money adoption, this approach generates policy-relevant insights that can inform the design of gender-sensitive financial inclusion initiatives and humanitarian cash-transfer programs in fragile state settings.


$$\begin{array}{c} {WI}_{hj}=\beta_{o}+\beta_{\text{1}}\hat{{AMMU}_{hj}}+\beta_{\text{2}}{Fragile}_{hj}+{\beta_{\text{3}}\left(\hat{{AMMU}_{hj}}\times {Fragile}_{hj}\right)+\gamma X}_{hj}+\varepsilon_{hj} \end{array}$$ (9)

where the dependent variable ${WI}_{hj}$ represents the Welfare Index, as defined earlier, for household h in community j. The main explanatory variable of interest, $\hat{{AMMU}_{hj}}$, is a binary indicator capturing whether the household is a predicted active mobile money user, as derived from the first-stage instrumental variable estimation. The model further controls for a set of household and individual characteristics, $X_{hj}$, including household size, age and education of the household head, rural or urban residence, employment status, monthly income, and gender of the household head, among others. Finally, $\varepsilon_{hj}$ is the error term, capturing unobserved factors affecting household welfare. This setup allows us to isolate not only the average welfare effect of mobile money adoption but also its differential impact in fragile state contexts, with particular relevance for policy targeting in regions where institutional weaknesses and recurrent shocks undermine conventional financial systems.

Table 10 reports results from a model assessing how active mobile money use, provincial fragility, and their interaction affect household welfare in Mozambique, controlling for key demographic and socioeconomic factors. The analysis tests whether mobile money delivers greater welfare benefits in provinces affected by conflict or extreme climatic events.

To further explore contextual heterogeneity, we distinguish between fragile and non-fragile provinces based on exposure to conflict and climate-related shocks. This classification reflects Mozambique’s significant regional variation, particularly in provinces affected by the Cabo Delgado insurgency and recurrent cyclones. Accordingly, we estimate a separate specification to assess whether the welfare effects of mobile money differ across these environments. This analysis is intended as a robustness and heterogeneity check rather than a new baseline model. The results, presented in Table 10, indicate that the welfare effects of active mobile money usage are more pronounced in fragile regions, consistent with its role in facilitating remittances, supporting consumption smoothing, and enhancing resilience under conditions of heightened uncertainty. Specifically, the interaction term (AMMU × Fragile Province) is positive and highly significant (β = 0.250, *p* < 0.01). This implies that in fragile provinces, households with active mobile money usage experience substantially greater welfare improvements compared to those in non-fragile provinces. When combining the direct effect of AMMU and its interaction with fragility, the total association amounts to an improvement of about 0.45 standard deviations in household welfare scores. In other words, mobile money delivers substantially larger welfare gains in areas where formal financial infrastructure is impaired. This amplified effect suggests that, in fragile contexts, mobile money becomes a critical resilience mechanism, facilitating remittances from displaced relatives, enabling humanitarian cash transfers, and supporting informal trade. Such outcomes mirror experiences documented in Somalia and Afghanistan, where mobile money has played a pivotal role in sustaining livelihoods under severe institutional and infrastructural disruption [54,62].

The average effect of active mobile money use in non-fragile provinces is both positive and statistically significant (β̂₁ = 0.200, p < 0.01). In practical terms, this means that, holding other factors constant, predicted active mobile money users enjoy welfare indices about 0.20 standard deviations higher than non-users. This result is consistent with evidence from Tanzania and Kenya, where mobile money has been shown to enhance household welfare through mechanisms such as remittance facilitation, income smoothing, and reduced transaction costs [58,59]. Therefore, even in relatively unstable environments, digital finance appears to provide meaningful improvements in living standards. provincial fragility is s negative and significant (β̂_2_ = −0.150, p < 0.05), indicating that households residing in fragile provinces have welfare levels around 0.15 standard deviations lower than those in non-fragile areas. This adverse effect reflects the disruptive effect of armed conflict, climatic shocks, displacement, and institutional breakdowns, as observed in other fragile settings such as South Sudan and Burundi [55; 64]. Consequently, fragility acts as a structural constraint on household welfare, limiting economic opportunities and undermining livelihoods.

Taken together, these findings emphasize that while mobile money benefits households broadly, its role is particularly transformative in fragile states, with heightened advantages for women-headed households who face overlapping vulnerabilities.

#### 4.4.2 Predicted household welfare by mobile money use and demographics.

**Fig 1:**
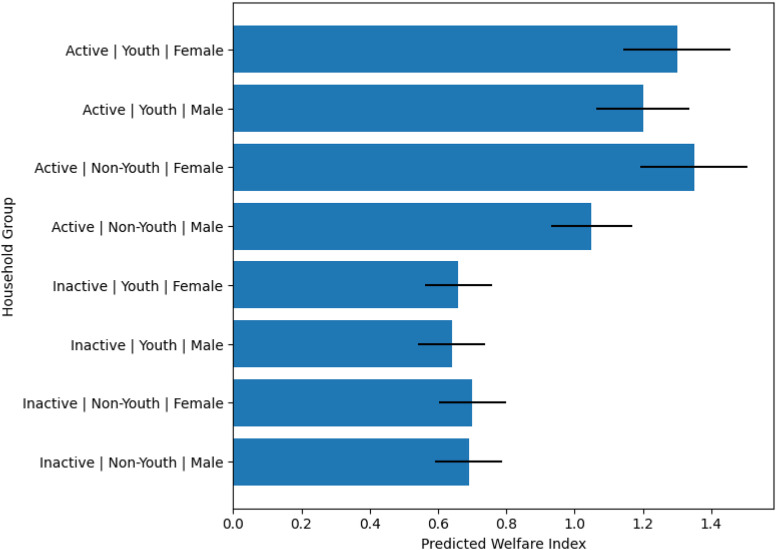
Predicted welfare index with 95% confidence intervals illustrating differences in household welfare across active mobile money users, dormant account holders, and non-users, and the estimated welfare effects of active mobile money use among women-, youth-, and male-headed households in Mozambique. Predicted values were obtained from the Instrumental Variable (IV) regression model using predictive margins, with 95% confidence intervals indicating the precision of the estimated welfare effects across the different categories of mobile money users and household groups.

Table 11 and Fig 1 highlights how household welfare varies by mobile money usage, youth status, and the gender of the household head. The results show that households actively using mobile money consistently exhibit higher welfare across all demographic groups compared to those who do not. For instance, non-youth, male-headed households without mobile money have a predicted welfare index of 0.661, whereas their counterparts who actively use mobile money record a significantly higher welfare index of 1.007. This finding supports existing evidence that mobile money improves household welfare through mechanisms such as remittance facilitation, savings mobilization, and reduced transaction costs [2,7]. Crucially, these welfare-enhancing effects are especially pronounced in fragile states like Mozambique, where formal financial institutions are often absent, inaccessible, or dysfunctional. In such settings, mobile money serves as a vital financial infrastructure, helping households manage risks, receive income, and smooth consumption in the face of institutional weakness and recurring economic or environmental shocks [64,56].

**Table 11:** 2SLS effects of mobile money on individual welfare components.

Variable	Own Food Consumption (0–2)	Health Expenditure (MZN)	Education Expenditure (MZN)
**Second Stage**			
Predicted Active Mobile Money User (ÂMMU)	–0.083** (0.033)	–0.88*** (0.28)	0.39** (0.19)
Predicted Dormant Mobile Money Account	–0.019 (0.031)	–0.56** (0.26)	0.14 (0.17)
ÂMMU × Female Head	–0.041 (0.028)	–0.44** (0.18)	0.21* (0.12)
ÂMMU × Youth Head	–0.036 (0.026)	–0.37* (0.20)	0.17 (0.13)
Institutional Effect (InstEff)	0.062** (0.025)	0.52*** (0.17)	0.48*** (0.15)
**Controls**			
Household Size	0.013 (0.010)	0.17 (0.15)	0.19 (0.14)
Household Head Age	0.001 (0.001)	0.01 (0.02)	0.01 (0.02)
Household Head Education (Years)	–0.010 (0.007)	0.29*** (0.09)	0.41*** (0.08)
Employment Status	–0.026 (0.017)	0.52** (0.23)	0.61** (0.21)
Monthly Income (log, MZN)	–0.039* (0.021)	0.65*** (0.19)	0.69*** (0.18)
Rural Residence	0.061** (0.026)	–0.71** (0.30)	–0.64** (0.27)
Province Fixed Effects	Yes	Yes	Yes
Observations (n)	900	900	900
R²	0.3	0.35	0.33

**Note:** Robust standard errors in parentheses. * p < 0.10, ** p < 0.05, *** p < 0.01. Estimates are based on the same 2SLS specification as Table 7.

**Source:** Results estimates.

In addition, youth-headed households demonstrate distinct welfare patterns that further reflect the dynamics of fragility. Without mobile money, youth-headed households record a welfare index of 0.605, lower than the 0.661 recorded for non-youth households, highlighting the economic marginalization and structural vulnerabilities youth face in fragile economies, such as high unemployment, limited assets, and exclusion from credit markets [18,60]. However, once these households adopt and actively use mobile money, their welfare rises significantly to 1.171, surpassing that of non-youth users (1.007). This finding reinforces the notion that digital financial services can compensate for institutional deficiencies by offering young people a means to engage in productive activities, receive remittances, and access emergency funds, even in the absence of traditional employment structures [59, 61].

Furthermore, female-headed households also benefit notably from mobile money adoption, particularly in fragile state contexts where women often experience disproportionate exclusion from formal economic and financial systems. Female-headed households that actively use mobile money report a welfare index of 1.137, higher than male-headed counterparts (1.007), while female youth-headed households exhibit the highest welfare index of 1.301. These findings are in line with research showing that digital finance disproportionately benefits women by enhancing economic autonomy, improving intrahousehold bargaining power, and facilitating access to gender-sensitive savings and credit tools [6,14]. In fragile settings, these effects are magnified, as women often serve as the primary managers of household welfare amid conflict, displacement, and climate shocks [52). Thus, mobile money emerges as a vital instrument for inclusive welfare enhancement in environments where conventional financial systems remain fragmented or inaccessible.

## 5. Policy implications

The findings demonstrate that active mobile money use, rather than account ownership alone, is associated with improved household welfare, particularly among women, youth, and households residing in fragile settings. Accordingly, policies should move beyond expanding financial access to fostering sustained and meaningful use of digital financial services. This distinction is especially important in fragile states such as Mozambique, where conflict, climate-related shocks, weak financial infrastructure, and institutional constraints continue to limit both financial inclusion and household resilience [13,14,56,62]. First, strengthening digital infrastructure remains a prerequisite for maximizing the welfare benefits of mobile money. Expanding mobile network coverage, increasing agent density, improving electricity reliability, and enhancing digital connectivity in conflict- and disaster-affected regions can reduce geographical barriers to financial inclusion where conventional banking services remain limited or absent [13,56]. However, infrastructure investments should be integrated into broader national digital transformation and financial inclusion strategies to ensure that expanded access translates into sustained socioeconomic gains rather than merely increasing the availability of financial services [3,64,56].

Nevertheless, infrastructure expansion alone is unlikely to generate meaningful welfare improvements. The results indicate that welfare gains arise primarily through active engagement with mobile money services, reinforcing evidence that effective financial inclusion depends not only on access but also on regular and informed usage [2,44,43,49]. Consequently, policymakers should complement infrastructure investments with digital and financial literacy programmes that strengthen users’ confidence, financial capability, and trust in digital financial systems. Targeted interventions, including simplified onboarding procedures, consumer protection mechanisms, and locally tailored financial education—are particularly important for women, youth, and rural households, who continue to face disproportionate barriers to digital financial participation [64,60]. Beyond promoting individual adoption, the findings also underscore the value of embedding mobile money within broader public policy frameworks. Integrating digital payment platforms into humanitarian assistance, social protection programmes, agricultural subsidies, and emergency cash transfer systems can improve the speed, transparency, and accountability of public transfers while reducing transaction costs and leakages, particularly during periods of conflict or climate-related crises [14,56,62]. Such integration would strengthen the responsiveness of social protection systems while enhancing household resilience to recurrent economic and environmental shocks.

Equally important, the empirical evidence highlights the need for gender-responsive digital financial policies. Expanding access to mobile-based savings, microcredit, insurance products, and digital savings groups can strengthen women’s economic resilience and improve the welfare of female-headed households [13,14]. However, these interventions are unlikely to achieve their full developmental potential unless accompanied by broader efforts to address persistent structural constraints, including limited digital literacy, unequal access to mobile devices, affordability challenges, and prevailing social norms that continue to restrict women’s participation in formal financial systems [6,7,13,64]. While these policy recommendations are strongly supported by the empirical findings, they should be interpreted alongside the study’s methodological limitations. Although the instrumental variable (IV) approach was employed to mitigate endogeneity arising from self-selection and potential reverse causality, its validity depends critically on the exclusion restriction, that the instrument effects household welfare solely through its influence on mobile money adoption and not through alternative channels [2,44,43]. Although this assumption is theoretically grounded and supported by the study’s identification strategy and empirical diagnostics, it cannot be verified directly using observational cross-sectional data. Consequently, the estimated effects should be interpreted as providing credible evidence consistent with a causal relationship rather than definitive proof of causality.

Furthermore, because the analysis relies on a single cross-sectional survey, it cannot capture dynamic behavioural adjustments or the longer-term welfare impacts of mobile money adoption. Future research using longitudinal datasets, panel designs, natural experiments, or randomized evaluations would strengthen causal identification and provide deeper insights into the persistence and evolution of digital financial inclusion effects over time [18,31,45]. Thus, the findings suggest that realizing the full developmental potential of mobile money requires coordinated investments in digital infrastructure, financial capability, institutional integration, and inclusive financial ecosystems. At the same time, recognizing the methodological constraints associated with cross-sectional observational data encourages a balanced interpretation of the results and identifies important priorities for future research. These considerations provide the foundation for the concluding section, which summarizes the study’s principal contributions and their implications for research, policy, and practice.

## 6. Conclusion

This study examined the impact of mobile money on household welfare in Mozambique, a fragile state facing recurrent climate shocks, governance challenges, and persistent poverty. Generally, the findings indicate that active mobile money use improves welfare, notably through reduced reliance on own food production, reflecting greater market integration and liquidity, as well as increased health-related expenditures. Importantly, these effects are particularly pronounced among youth- and female-headed households, who face greater barriers to formal financial access. In contrast, dormant accounts have no statistically significant effect on welfare, underscoring that access alone is insufficient and that active use is the key driver of welfare gains. However, significant spatial inequalities persist, as rural and conflict-affected regions exhibit lower welfare outcomes despite mobile money access, indicating that structural constraints limit its full impact. Therefore, policies should prioritize gender-sensitive financial literacy, expand rural agent networks, integrate mobile money into social protection systems, and strengthen regulatory frameworks. In addition, improving digital infrastructure is essential. In addition, policies should explicitly address the mobile gender gap by promoting affordable handset ownership, digital skills, and women’s access to mobile internet and financial services [37]. Such interventions would complement broader efforts to expand digital financial inclusion and reduce poverty across vulnerable populations [66,67]. Finally, given the cross-sectional nature of the data, causal interpretation remains subject to limitations. Accordingly, future research should prioritize the use of panel data or natural experimental designs to strengthen causal identification and better capture the dynamic effects of mobile money on household welfare over time.
